# Serious conditions among patients with non-specific chief complaints in the pre-hospital setting: a retrospective cohort study

**DOI:** 10.1186/s13049-020-00767-0

**Published:** 2020-07-29

**Authors:** Robert Ivic, Lisa Kurland, Veronica Vicente, Maaret Castrén, Katarina Bohm

**Affiliations:** 1 Karolinska Institute, Department of Clinical Science and Education, Södersjukhuset, Stockholm, Sweden; 2 Academic Emergency Medical Service, Region Stockholm, Stockholm, Sweden; 3 Department of Medical Sciences and Department of Emergency Medicine, Örebro University, Örebro, Sweden; 4 Emergency Medicine, Helsinki University and Department of Emergency Medicine and Services, Helsinki University Hospital, Helsinki, Finland; 5 Department of Emergency Medicine, Södersjukhuset, Stockholm, Sweden

**Keywords:** Pre-hospital emergency care, Non-specific complaints, Emergency medical service

## Abstract

**Background:**

Emergency Medical Services (EMS) are faced daily with patients presenting with a non-specific chief complaints (NSC); i.e. decreased general condition, general malaise, sense of illness, or just being unable to cope with usual daily activities. Patients presenting with NSCs often have normal vital signs. It has previously been established that however, NSCs may have a serious underlying condition that has yet to be identified. The primary outcome of this study was to determine the prevalence of serious conditions in patients presenting with NSCs to the EMS.

**Method:**

A retrospective cohort study of patients ≥18 years of age who were reported as presenting with chief complaints compatible with NSCs to the EMS in Stockholm Region and transported to an emergency department between January 1st, 2013 and December 31st, 2013. Patients were identified via the EMS electronic health care record and followed via records from the National Patient Registry and Causes of Death Registry at Sweden’s National Board for Health and Welfare. The definition of serious condition was defined by expert consensus. Descriptive statistics as well as regression analyses were used.

**Results:**

A total of 3780 patients were included, with a median age of 77 years. A serious condition was present in 35.3% of the patients. The in-hospital mortality rate for the group with serious conditions was 10.1% (OR 6.8, CI 95%, 4.1–11.3), and the 30-day mortality rate was 20.2% (OR 3.1, CI 95%, 2.3–4.0). In the group with no serious conditions the rates were 1.0 and 4.2%, respectively. The total hospitalization rate was 67.6%. The presence of serious conditions as well as increased mortality rates were associated with Rapid Emergency Triage and Treatment system (RETTS) as well as National Early Warning Score (NEWS) irrespective of triage score.

**Conclusion:**

More than one-third of the patients presenting with NSCs to EMS had a serious underlying condition which was associated with increased mortality and hospitalization rates.

**Trial registration:**

Not applicable.

## Background

Emergency Medical Services (EMS) play an important role in assessing, initiating treatment and transporting patients to the Emergency Department (ED). Patients present to the EMS with a primary symptom, i.e. chief complaint. Chief complaints without specific components linked to anatomical, clear physiological and pathophysiological systems are considered as non-specific chief complaints. Patients are assessed based on vital signs and patient history according to local EMS guidelines [1]. The number of patients presenting to the EMS with NSCs is unknown, as is the prevalence of serious conditions in this group. Although, non-specific chief complaints (NSCs) have been studied in the pre-hospital setting [2, 3], it has been shown, however, that up to one in every five patients in the ED has a NSC [4]. Previous studies have shown that half of these patients are suffering from a serious underlying condition [4, 5]. Patients presenting with NSCs often present as “affected general health condition” or “decreased general condition,” “general malaise,” “sense of illness” or “just being unable to cope with usual daily activities,” and often present with normal vital signs [6–8]. Patients presenting with NSCs are often elderly, and as many as half of these patients suffer from an acute condition [9]. The elderly presenting with NSCs are often under-triaged [9], despite having the highest in-hospital mortality rates of all patients with non-trauma/non-surgical chief complaints in the ED [10]. Therefore, the primary aim of the current study was to establish the prevalence of serious conditions among patients presenting to EMS with NSCs. The secondary aim was to determine the mortality rates for patients presenting with NSCs.

## Methods

### Study design

A retrospective cohort of adult patients presenting to the EMS in Stockholm Region, Sweden with a chief complaint compatible with NSC, according to the definition below, between January 1st, 2013 and December 31st, 2013 were included in the study.

### Study setting and population

Stockholm Region had a population of approximately 2.1 million (as of 2015). The Stockholm Region is responsible for operating the EMS and in this context relate to ambulance services. EMS services are provided by AISAB owned by the region [11], and two private companies [12, 13]. AISAB, performs approximately 42% of the total of 190,000 annual ambulance assignments in the Region. The Stockholm Region’s ambulance assignments are distributed between 71 ambulances, 31 of which are operated by AISAB. All ambulances are staffed by a nurse specialist in prehospital care and an emergency medical technician (EMT).

### Study material

All patients presenting with NSCs, according to the definition below, to the EMS delivered by AISAB were included in the current study. Inclusion criteria were: all patients ≥18 years whose EMS record contains a presenting complaint of “decreased general condition,” “fatigue,” “malaise” or “feeling unwell” according to the electronic health care record (eHR), and who were subsequently transported to an ED. The exclusion criteria were duplicated records, referrals, non-conveyance to an ED or patients deceased during the assignment.

### Definition of serious condition

The definition of serious conditions established initially developed for ED purposes by Nemec et al. [5] was adapted to the EMS by “translating” the list of serious conditions into ICD-10 diagnosis codes including sub-codes. Chronic diagnoses and codes corresponding to non-acute diagnoses listed in the original Nemec et al. publication [5] were excluded from the list of ICD-10 diagnosis codes applied to the current EMS based study. Additional adaptations were made as follows: although neoplasms are by definition serious, neoplasms were not considered serious in the EMS context unless the patient was admitted to in-hospital care or died within 30 days of index EMS assessment. Infectious diseases were considered a serious condition if the patient was admitted to in-hospital care. The modified definition of serious condition was based on expert consensus, all of whom are senior emergency medicine- physicians with extensive prehospital experience or experienced emergency department and nurse specialists in prehospital care (Additional file 1).

### Data collection

**Fig. 1:**
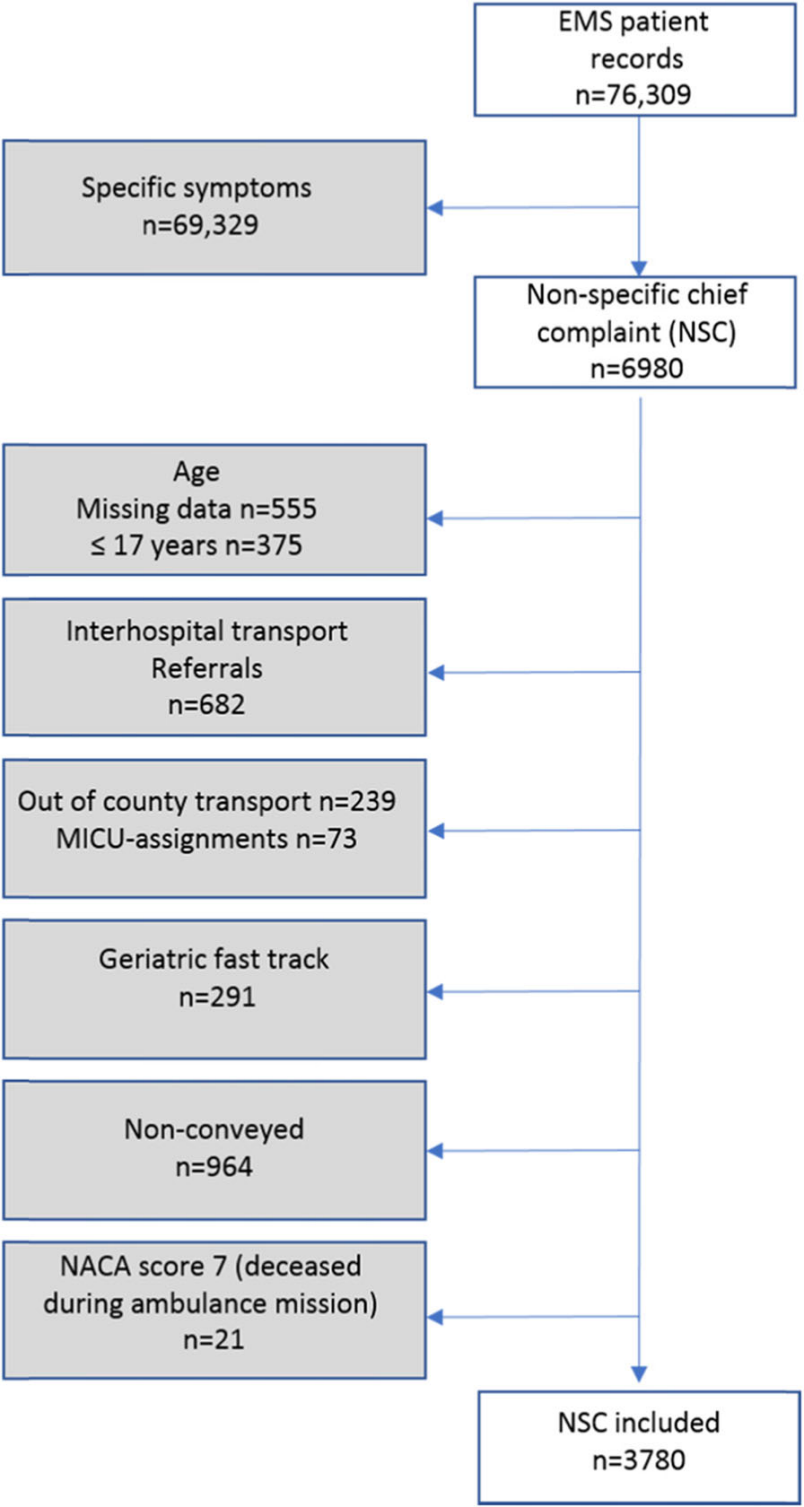
Flowchart of inclusion and exclusion criteria. EMS: emergency medical services; NSC: non-specific complaints; NACA: National Advisory Committee for Aeronautics

We identified the NSCs using the CAK-net eHR used by the EMS [14]. CAK-net is built on a maximum of three primary assessment categories, the first of which is mandatory. Three of the categories are non-specific: “decreased general health condition,” “general, unspecific,” and “undefined symptoms.” All other categories were excluded since they relate to specific presentations. We applied the NSC categories on all three primary assessment categories in the CAK-net database for the time-period of the study. The exclusion criteria were applied manually after the first acquisition of patients assessed as NSC (Fig. 1).

Patient data was obtained from CAK-net (age, sex, vital signs at EMS triage, Glasgow Coma Scale [GCS], National Advisory Committee for Aeronautics [NACA] score), the National Patient Register at Sweden’s National Board of Health and Welfare (ICD-10 code at ED discharge, ED disposition [release to home or hospital admission], in-hospital length of stay [LOS], ICD-10 code from in-hospital discharge) as well as mortality rates from the Causes of Death register at the National Board of Health and Welfare.

Triage levels were calculated retrospectively from vital signs from the index EMS assessment and based on RETTS (rapid emergency triage and treatment score), the predominant triage system in EMS in Sweden today, and NEWS (national early warning score). RETTS is a five-level triage scale based on cut-off levels for vital signs and 59 chief complaint algorithms called emergency symptoms and signs (ESS) [15]. The ESS were not included in the retrospective calculation of RETTS. The triage levels are indicated by color—blue, green, yellow, orange and red—with blue being the least urgent, and red the most urgent. RETTS’ lowest level (blue) is not used by the EMS which makes the green level the least urgent in the EMS-setting. The NEWS scoring system is based on vital sign categories with the aggregated score converted to a three-level scale of clinical risk: low (0–4), medium (5–6) and high (≥7) [16–18].

The Charlson comorbidity index (CCI) was calculated for each patient. All comorbid diseases not yet completely resolved were recorded (19).

### Data analysis

Descriptive statistics were used. Differences between groups were evaluated using a Chi^2^-test for categorical variables and a Mann-Whitney U Test for numerical variables. Logistic regression analysis was performed to assess the association of individual risk factors such as RETTS and NEWS triage scores with the primary outcome (presence of a serious condition) as well the secondary outcomes (24-h, 30-day and in-hospital mortality rates) after the index assessment by the EMS. The lowest, i.e. green RETTS triage level and low clinical risk according to NEWS were analyzed separately in a stratified model. The results are presented as odds ratios (OR) within a 95% confidence interval (CI). All statistical analysis was performed using IBM SPSS Statistics for Windows, version 25 (IBM Corp. Armonk, New York, USA).

### Ethical considerations

The study was approved by the Regional Ethic Committee in Stockholm, Sweden (reg. no. 2014/1999–31/4; 2016/1724–32).

## Results

A total of 3780 patients with NSCs were included in the current study (Fig. 1). The median age was 77 years of age. Triage levels were: 60.8% (*n* = 2027) were green according to RETTS and 76.3% (*n* = 2845) had low clinical risk according to NEWS. The overall admission rate to in-hospital care was 67.6% (*n* = 2557) with a median in-hospital LOS of 5 days (range 0–72 days). The median CCI score was 1 point (range 0–9 points) (Table 1).

**Table 1:** Baseline characteristics for patients presenting with NSCs to the EMS

		Total			Serious condition			Serious condition			*p* value.
					present			not present			
		*N* = 3780			*n =* 1334 (35.3%)			*n* = 2446 (64.7%)			
		n	Md	(%)	n	Md	(%)	n	Md	(%)	
Sex	Female	2033		(53.8)	682		(51.1)	1351		(55.2)	0.015
	Male	1747		(46.2)	652		(48.9)	1095		(44.8)	
Age			77			83			72		< 0.001
GCS	13–15	712		(18.8)	270		(20.2)	442		(18.1)	0.005
	9–12	60		(1.6)	32		(2.4)	28		(1.1)	
	≤ 8	36		(1.0)	15		(1.1)	21		(0.9)	
	Missing	2972		(78.6)	1017		(76.2)	1955		(79.9)	
NACA	0–2	765		(20.2)	140		(10.5)	625		(25.6)	< 0.001
	3–4	2786		(73.4)	1104		(82.8)	1682		(68.8)	
	5–7	76		(2.0)	44		(3.3)	32		(1.3)	
	Missing	153		(4.0)	46		(3.4)	107		(4.4)	
RETTS	Green	2027		(60.8)	484		(40.7)	1543		(71.9)	< 0.001
	Yellow	677		(20.3)	330		(27.8)	347		(16.2)	
	Orange	418		(12.5)	241		(20.3)	177		(8.2)	
	Red	214		(6.4)	134		(11.3)	80		(3.7)	
NEWS	Low risk	2845		(76.3)	804		(61.0)	2041		(84.7)	< 0.001
	Medium risk	446		(12.0)	230		(17.5)	216		(9.0)	
	High risk	438		(11.7)	284		(21.5)	154		(6.4)	
CCI	Md		1			2			1		< 0.001
Admitted	Yes	2557		(67.6)	1334		(100)	1223		(50)	< 0.001
	No	1223		(32.4)	0		(0)	1223		(50)	
In-hospital LOS			5			6			3		< 0.001
In-hospital mortality		160		(4.2)	135		(10.1)	25		(1.0)	< 0.001
24 h mortality		42		(1.1)	26		(1.9)	16		(0.7)	< 0.001
30 day mortality		372		(9.8)	269		(20.2)	103		(4.2)	< 0.001

*RETTS* Rapid Emergency Triage and Treatment System, *NEWS* national early warning score, *CCI* Charlson comorbidity index, *LOS* length of stay

*Differences between serious conditions present/not present expressed as p-values.*

A serious condition was present in 35.3% (*n* = 1334) of the patients presenting with NSCs.

Patients with serious conditions presented with higher triage levels according to both the RETTS and NEWS as compared to patients with no serious conditions (Table 1).

Overall in-hospital mortality was 4.2% (*n* = 160), 24 h mortality 1.1% (*n* = 42), and 30-day mortality was 9.8% (*n* = 372). In the group with serious conditions, in-hospital mortality was 10.1% (*n* = 135) (OR 6.8, CI 95%, 4.1–11.3), 24 h mortality 1.9% (*n* = 26), and 30-day mortality was 20.2% (*n* = 269) (OR 3.1, CI 95%, 2.3–4.0). In the group with no serious conditions the corresponding in-hospital mortality was 1.0% (*n* = 25), 24 h mortality 0.7% (*n =* 16), and 30 day mortality 4.2% (*n* = 103) (Tables 1 and 2).

**Table 2:** Logistic regression for serious conditions and mortality rates

		Serious condition		24 h Mortality		In-hospital mortality		30-day mortality	
		OR	95% CI	OR	95% CI	OR	95% CI	OR	95% CI
Serious condition	Yes	–	–	1.6	0.7–3.7	6.8*	4.1–11.3	3.1*	2.3–4.0
	No	–	–	0.6	0.2–2.4	0.1*	0.1–0.2	0.3*	0.2–0.4
RETTS-vs	Green	ref		ref		ref		ref	
	Yellow	1.9*	1.6–2.3	0.8	0.2–3.2	1.3	0.6–2.7	1.4	1.0–2.0
	Orange	2.5*	1.9–3.2	2.5	0.7–9.0	1.7	0.8–3.7	2.7*	1.8–3.9
	Red	2.6*	1.8–3.7	9.1*	2.5–33.4	3.1*	1.3–7.8	3.5*	2.2–5.6
NEWS	Low risk	ref		ref		ref		ref	
	Medium risk	1.6*	1.3–2.1	2.3	0.7–7.2	1.0	0.5–2.2	1.5*	1.0–2.1
	High risk	2.3*	1.7–3.0	1.5	0.5–5.0	0.6	0.2–1.6	1.7*	1.2–2.4

*Regression model adjusted for sex, age, NEWS and RETTS.*

*NEWS* national early warning score, *RETTS-vs* Rapid Emergency Triage and Treatment System – vital signs.

* p < 0.05

Stratified models of triage levels are presented in Table 3. In RETTS green (*n* = 2027) and NEWS low clinical risk (*n* = 2845), a serious condition was present in 23.9% (*n* = 484) and 28.3% (*n* = 804) of the patients respectively. Thirty day mortality was 13.0% (*n* = 63) (OR 5.0, CI 95%, 3.2–7.9) and 14.1% (*n* = 113) (OR 3.7, CI 95%, 2.7–5.2) respectively in the group with serious conditions.

**Table 3:** Logistic regression for serious conditions and mortality rates

			24 h Mortality		In-hospital mortality		30-day mortality	
			OR	95% CI	OR	95% CI	OR	95% CI
RETTS Green	Serious condition	Yes	9.1*	1.0–78.3	15.6*	6.0–40.6	5.0*	3.2–7.9
		No	0.1*	0.0–1.0	0.1*	0.0–0.2	0.2	0.1–0.3
NEWS Low clinical risk	Serious condition	Yes	3.0	0.9–10.0	10.5*	5.2–21.0	3.7*	2.7–7.9
		No	0.3	0.1–1.1	0.1*	0.0–0.2	0.3*	0.1–0.3

*Regression model adjusted for sex, age, NEWS and RETTS.*

*NEWS* national early warning score*, RETTS-*vs Rapid Emergency Triage and Treatment System – vital signs.

* p < 0.05

The most common discharge diagnosis for patients with serious conditions was infectious disease (31.0%, *n* = 413), followed by cardiovascular disease (26.4%, *n* = 352) (Table 4).

**Table 4:** Distribution of disease groups by discharge diagnosis (ED, in-hospital) among patients with serious conditions in the group of patients presenting to the EMS with NSCs

	Total	
	*n* = 1334	
Diagnosis group	n	(%)
Infectious	413	(31.0)
Cardiovascular	352	(26.4)
Neurological	148	(11.1)
Neoplasms	130	(9.7)
Endocrine, nutritional and metabolic	97	(7.3)
Mental or behavioral	64	(4.8)
Pulmonary	51	(3.8)
Nephrology/Urology	47	(3.5)
Abdominal	32	(2.4)
Poisoning	0	(0.0)
Blood, blood-forming organs, immune system	0	(0.0)

## Discussion

The results show that more than one-third of patients with NSCs had a serious condition. Mortality rates were almost four times higher in the group with serious conditions as compared to those without serious conditions.

More than one-third of the patients presenting with NSCs in the current study had a serious condition as compared with approximately 60% in prior studies in ED settings [5, 19, 20]. It is however, surprising that the prevalence of serious conditions in the current study was lower compared to studies of patients presenting to the ED with NSCs without concern to mode of arrival [5, 19, 20]. This is in conflict with, patients arriving to the ED by ambulance are in general sicker than those who “walk in” [21]. The definition of serious conditions in the current study may partly explain the difference since it may select for a sicker population. This lower prevalence might also be explained in part by the assessments made by the EMS personnel, if the patient is perceived as more ill the assessment by the EMS may tend to use a more specific complaint. Despite these differences, the prevalence of serious conditions among patients with NSCs is high and should be taken into consideration in clinical practice. With that in mind, the retrospective triage scores according to RETTS and NEWS were as expected associated with the prevalence of serious conditions when patients were assessed to a higher triage level. Nevertheless, the majority of patients with NSCs had low triage levels. Interestingly, patients with low triage scores had nearly as often prevalent serious conditions comparable to the current studies overall population. This implies that triage levels are an insufficient tool with which to identify serious conditions. We suggest that the reason for this is likely to be that triage levels are based on vital signs. Patients with NSCs were in general elderly, which is consistent with prior research [2, 5, 10, 19, 22]. Almost half the patients in the current study were over 80 years old, and the majority presented with vital signs within the normal range. The presence of normal vital signs can likely be explained by the physiological changes in older adults, which tend to reduce the ability of organ systems to adapt to physiological changes, leading to an absence of deranged vital signs [7, 19, 23]. How to identify serious conditions other than by triage level requires further investigation.

Mortality rates were higher in the group with serious conditions as compared to those with no serious conditions, among patients presenting with NSCs to the EMS. The mortality rate in the current study was slightly higher than the mortality rate in the study by Nemec et al. [5], but not as high as the rates reported by Säfwenberg et al. [10]. The higher mortality rate in comparison to the current study could be discussed, since Säfwenberg et al. [10] only studied patients with one of the NSCs, i.e. general disability, which may be selecting for a sicker population. Both overall in-hospital mortality rates and mortality rates for patients with serious conditions were comparable with those in previous studies [2, 10, 24]. Wallgren et al. [25] reported in-hospital mortality rates as high as one third of the patients with sepsis and a presenting complaint of decreased general condition (an NSC), while non-septic patients with decreased general condition had one tenth of that mortality. That one in every five patients in the current study presenting with NSCs and having a serious condition died within 30 days underscores the need to change our management of this patient population. In conclusion, and based on the current results, triage systems based on vital signs appear to be insufficient both in identifying serious conditions and in predicting mortality among patients with non-specific chief complaints.

The most common discharge diagnosis was an infectious disease, such as pneumonia or urinary tract infection. The infectious diseases were followed by cardiovascular and neurological diseases. The clinical picture at the index EMS assessment can be non-specific and more sophisticated diagnostic tools may be required to establish the diagnosis.

Idenitfication of patients with serious conditions among those with NSC in the prehospital setting remains a challenge. Point of care tests of biomarkers may aid in the identification of single diagnosis time critical conditions e.g. myocardial infarctions and sepsis in this group, and may be of some use. However, this will likely leave a larger proportion of the patients with NSC remaining as undifferentiated. In this case we believe that two important approaches need to be considered. One is the advancement of experienced clinicians in the field with the skill and knowledge to make clinical decisions based on the complex background of individual patients. The other is on mutual terms to include the patients’ own wish for further care, i.e. a patient-centered approach. Also this requires experienced clinicians in field.

### Methodological considerations and limitations

Since there is no universal definition of serious conditions, the authors chose to modify a previous definition [5]. The Swedish National Board for Health and Welfare’s National Patient Register is based on registered ICD-10 codes and not assessed conditions by EMS. Therefore, it was necessary to translate the list of serious conditions into ICD-10 codes. Since conditions may have both acute and chronic components. We chose admission to in-hospital care as a proxy for a condition to be “serious” in some of the listed conditions. Our definition of serious conditions and their translation into ICD-10 codes may be more precisely transferrable in future research.

Another limitation was related to the CAK-net electronic records system used by the EMS, which made it challenging to extract patient data based on the three primary assessment classifications. The ability of EMS personnel to document patients with NSCs using different assessment categories may have led to some patients being excluded from the data. Despite this, the size of the current cohort is relatively large and the results therefore considered to be generalizable.

Triage scores were calculated retrospectively based on the vital signs alone, i.e. not the emergency signs and symptoms typically required for a complete triage score according to RETTS. It is unlikely, however, that this additional information would alter the triage level, since the signs and symptoms in the studied population are non-specific which have inherently low triage scores in RETTS. The risk of under-triage in patients presenting with NSCs is evidenced by the increased risk of in-hospital mortality [2].

## Conclusions

The results show that more than one in three of the patients presenting to the EMS with NSC have a serious condition. The presence of serious conditions is associated with both a tenfold increase in-hospital mortality and a five times higher 30-day mortality compared to patients without serious conditions. Low triage scores are common in patients presenting to EMS with NSCs despite underlying serious conditions and high mortality. Hence, vital-sign-based triage systems appear to be insufficient when attempting to identify patients at high risk of having a serious condition and of dying among those presenting to EMS with non-specific chief complaints.

## Supplementary information

**Additional file 1.** List of serious conditions.

## Data Availability

Anonymized data analyzed for the current study will be shared if a reasonable request is made by a qualified investigator to the corresponding author.
