# Supplementary material for: Serious conditions among patients with non-specific chief complaints in the pre-hospital setting: a retrospective cohort study
Source: Scand J Trauma Resusc Emerg Med. 2020 Jul 29;28:74. doi: 10.1186/s13049-020-00767-0 (PMC7391698; doi:10.1186/s13049-020-00767-0)
Supplement: Supplementary file 1 — Additional file 1. List of serious conditions. [file 13049_2020_767_MOESM1_ESM.docx]

***Appendix 1: List of serious conditions***

| **System** | **Diagnosis** | **ICD-10** |
| --- | --- | --- |
| **Cardiovascular** | | |
| Congestive heart failure | Right ventricular failure | I50.0 |
|  | Left ventricular failure | I50.1 |
|  | Heart failure, unspecified | I50.9 |
| Acute coronary syndrome | Acute myocardial infarction, unspecified | I21.9 |
|  | Unstable angina | I20.0 |
|  | Acute transmural myocardial infarction of other sites | I21.2 |
|  | Acute transmural myocardial infarction of unspecified site | I21.3 |
|  | Acute ischaemic heart disease, unspecified | I24.9 |
| Aneurysm and dissection | Dissection of aorta [any part] | I71.0 |
|  | Aneurysm and dissection of carotid artery | I72.0 |
|  | Aneurysm and dissection of iliac artery | I72.3 |
|  | Aneurysm and dissection of artery of lower extremity | I72.4 |
|  | Aneurysm and dissection of other precerebral arteries | I72.5 |
|  | neurysm and dissection of vertebral artery | I72.6 |
|  | Aneurysm and dissection of other specified arteries | I72.8 |
|  | Aneurysm and dissection of unspecified site | I72.9 |
| Embolism | Pulmonary embolism with mention of acute cor pulmonale | I26.0 |
|  | Pulmonary embolism without mention of acute cor pulmonale | I26.9 |
|  | Embolism and thrombosis of abdominal aorta | I74.0 |
|  | Embolism and thrombosis of other and unspecified parts of aorta | I74.1 |
|  | Embolism and thrombosis of arteries of upper extremities | I74.2 |
|  | Embolism and thrombosis of arteries of lower extremities | I74.3 |
|  | Embolism and thrombosis of iliac artery | I74.5 |
| Peri/myocarditis | Acute nonspecific idiopathic pericarditis | I30.0 |
|  | Infective pericarditis | I30.1 |
|  | Other forms of acute pericarditis | I30.8 |
|  | Acute pericarditis, unspecified | I30.9 |
|  | Infective myocarditis | I40.0 |
|  | Other acute myocarditis | I40.8 |
|  | Acute myocarditis, unspecified | I40.9 |
| **Pulmonary** | | |
| Interstitial pulmonary disease | Acute drug-induced interstitial lung disorders | J70.2 |
|  | Chronic obstructive pulmonary disease with acute exacerbation, unspecified | J44.1 |
|  | Acute respiratory failure | J96.0 |
| **Abdominal** | | |
| Acute abdomen | Acute appendicitis with generalized peritonitis | K35.2 |
|  | Acute appendicitis with localized peritonitis | K35.3 |
|  | Acute peritonitis | K65.0 |
|  | Disorders of peritoneum in infectious diseases classified elsewhere | K67* |
|  | Acute parametritis and pelvic cellulitis | N73.0 |
|  | Female acute pelvic peritonitis | N73.3 |
| Hernias | Bilateral inguinal hernia, with obstruction, without gangrene | K40.0 |
|  | Bilateral inguinal hernia, with gangrene | K40.1 |
|  | Unilateral or unspecified inguinal hernia, with obstruction, without gangrene | K40.3 |
|  | Unilateral or unspecified inguinal hernia, with gangrene | K40.4 |
|  | Bilateral femoral hernia, with obstruction, without gangrene | K41.0 |
|  | Bilateral femoral hernia, with gangrene | K41.1 |
|  | Unilateral or unspecified femoral hernia, with obstruction, without gangrene | K41.3 |
|  | Unilateral or unspecified femoral hernia, with gangrene | K41.4 |
|  | Umbilical hernia with obstruction, without gangrene | K42.0 |
|  | Umbilical hernia with gangrene | K42.1 |
|  | Incisional hernia with obstruction, without gangrene | K43.0 |
|  | Incisional hernia with gangrene | K43.1 |
|  | Parastomal hernia with obstruction, without gangrene | K43.3 |
|  | Parastomal hernia with gangrene | K43.4 |
|  | Other and unspecified ventral hernia with obstruction without gangrene | K43.6 |
|  | Other and unspecified ventral hernia with gangrene | K43.7 |
| Gastrointestinal haemorrhage | Haematemesis | K92.0 |
|  | Melaena | K92.1 |
|  | Gastrointestinal haemorrhage, unspecified | K92.2 |
|  | Ulcer of oesophagus | K22.1 |
|  | Perforation of oesophagus | K22.3 |
|  | Gastric ulcer, acute with haemorrhage | K25.0 |
|  | Gastric ulcer, acute with perforation | K25.1 |
|  | Gastric ulcer, acute with both haemmorage and perforation | K25.2 |
|  | Duodenal ulcer, acute with haemorrhage | K26.0 |
|  | Duodenal ulcer, acute with perforation | K26.1 |
|  | Duodenal ulcer, acute with both haemmorage and perforation | K26.2 |
|  | Perforation of intestine (nontraumatic) | K63.1 |
|  | Peptic ulcer, site unspecified, acute with haemorrhage | K27.0 |
|  | Peptic ulcer, site unspecified, acute with perforation | K27.1 |
|  | Peptic ulcer, site unspecified, acute with both haemmorage and perforation | K27.2 |
|  | Gastrojejunal ulcer, acute with haemorrhage | K28.0 |
|  | Gastrojejunal ulcer, acute with perforation | K28.1 |
|  | Gastrojejunal ulcer, acute with both haemmorage and perforation | K28.2 |
|  | Acute haemorrhagic gastritis | K29.0 |
| **Neurological** | | |
| Cerebrovascular – ischemic | Cerebral infarction | I63* |
| Cerebrovascular – haemmorrhagic | Subarachnoid haemorrhage | I60* |
|  | Intracerebral haemorrhage | I61* |
|  | Other nontraumatic intracranial haemorrhage | I62* |
| Brain abscess/infection | Amoebic brain abscess | A06.6 |
|  | Phaeomycotic brain abscess | B43.1 |
|  | Tuberculous meningitis | A17.0 |
|  | Plague meningitis | A20.3 |
|  | Listerial meningitis and meningoencephalitis | A32.1 |
|  | Meningococcal meningitis | A39.0 |
|  | Viral meningitis | A87* |
|  | Herpesviral meningitis | B00.3 |
|  | Varicella meningitis | B01.0 |
|  | Varicella encephalitis | B01.1 |
|  | Zoster encephalitis | B02.0 |
|  | Zoster meningitis | B02.1 |
|  | Measles complicated by encephalitis | B05.0 |
|  | Measles complicated by meningitis | B05.1 |
|  | Mumps meningitis | B26.1 |
|  | Candidal meningitis | B37.5 |
|  | Bacterial meningitis | G00* |
|  | Meningitis in bacterial diseases classified elsewhere | G01* |
|  | Meningitis in other infectious and parasitic diseases classified elsewhere | G02* |
|  | Meningitis due to other and unspecified causes | G03* |
| Epilepsy | Status epilepticus | G41* |
| Inflammatory neuropathy | Guillain-Barré syndrome | G61.0 |
| **Infectious** | | |
| Pneumonia (only if admitted to hospital care) | HIV disease resulting in Pneumocystis jirovecii pneumonia | B20.6 |
|  | HIV disease resulting in lymphoid interstitial pneumonitis | B22.1 |
|  | Cytomegaloviral pneumonitis | B25.0 |
|  | Influenza with pneumonia, seasonal influenza virus identified | J10.0 |
|  | Influenza with other respiratory manifestations, seasonal influenza virus identified | J10.1 |
|  | Influenza with pneumonia, virus not identified | J11.0 |
|  | Influenza with other respiratory manifestations, virus not identified | J11.1 |
|  | Viral pneumonia | J12* |
|  | Pneumonia due to Streptococcus pneumoniae | J13* |
|  | Pneumonia due to Haemophilus influenzae | J14* |
|  | Bacterial pneumonia | J15* |
|  | Pneumonia due to other infectious organisms | J16* |
|  | Pneumonia in diseases classified elsewhere | J17* |
|  | Pneumonia, organism unspecified | J18* |
|  | Acute bronchitis | J20* |
|  | Hypersensitivity pneumonitis due to organic dust | J67* |
|  | Respiratory conditions due to inhalation of chemicals, gases, fumes and vapours | J68* |
|  | Pneumonitis due to solids and liquids | J69* |
|  | Abscess of lung with pneumonia | J85.1 |
| Urinary tract infection | Urinary tract infection, site not specified | N39.0 |
| Cholecystitis | Acute cholecystitis | K81.0 |
|  | Calculus of gallbladder with acute cholecystitis | K80.0 |
| Necrotizing fasciitis | Necrotizing fasciitis | M72.6 |
| Sepsis | Salmonella sepsis | A02.1 |
|  | Anthrax sepsis | A22.7 |
|  | Listerial sepsis | A32.7 |
|  | Candidal sepsis | B37.7 |
|  | Acute meningococcaemia | A39.2 |
|  | Streptococcal sepsis | A40* |
|  | Other sepsis | A41* |
|  | Actinomycotic sepsis | A42.7 |
|  | Toxic shock syndrome | A48.3 |
| Septic arthritis | Pyogenic arthritis | M00* |
| **Endocrine, nutritional and metabolic diseases** | | |
|  | Hypo-osmolality and hyponatraemia | E87.1 |
|  | Hyperkalaemia | E87.5 |
|  | Hypokalaemia | E87.6 |
|  | Addisonian crisis | E27.2 |
|  | Nondiabetic hypoglycaemic coma | E15* |
|  | Other disorders of pancreatic internal secretion | E16* |
|  | Hyperglycaemia, unspecified | R73.9 |
| **Nephrology/ urology** | | |
|  | Retention of urine | R33* |
|  | Acute renal failure | N17* |
| **Poisoning/ intoxication** (only if admitted to hospital care) | | |
|  | Botulism | A05.1 |
|  | Accidental poisoning by and exposure to nonopioid analgesics, antipyretics and antirheumatics | X40* |
|  | Accidental poisoning by and exposure to antiepileptic, sedative-hypnotic, antiparkinsonism and psychotropic drugs, not elsewhere classified | X41* |
|  | Accidental poisoning by and exposure to narcotics and psychodysleptics [hallucinogens], not elsewhere classified | X42* |
|  | Accidental poisoning by and exposure to other drugs acting on the autonomic nervous system | X43* |
|  | Accidental poisoning by and exposure to other and unspecified drugs, medicaments and biological substances | X44* |
|  | Accidental poisoning by and exposure to alcohol | X45* |
|  | Accidental poisoning by and exposure to organic solvents and halogenated hydrocarbons and their vapours | X46* |
| **Neoplasms** | | |
|  | All new, acute complications, all deaths within 30 days | C01-C26*  C31-C34*  C37-C41*  C43-C58*  C60-C86*  C90-C97* |
|  | Neoplasm in situ | D00-D09* |
